# Prediction of glycosylated hemoglobin level in patients with cardiovascular diseases and type 2 diabetes mellitus with respect to anti-diabetic medication

**DOI:** 10.3389/fendo.2024.1305640

**Published:** 2024-04-04

**Authors:** Alisher Ikramov, Shakhnoza Mukhtarova, Raisa Trigulova, Dilnoza Alimova, Saodat Abdullaeva

**Affiliations:** ^1^ Department of Mathematics, New Uzbekistan University, Tashkent, Uzbekistan; ^2^ Department of Applied Mathematics and Information Security, National University of Uzbekistan, Tashkent, Uzbekistan; ^3^ Laboratory of Biomedinformatics, Institute of Mathematics, Tashkent, Uzbekistan; ^4^ Laboratory of Cardiodiabetes, Republican Specialized Scientific and Practical Medical Center for Cardiology, Tashkent, Uzbekistan

**Keywords:** glycosylated hemoglobin, metformin, diabetes mellitus, random forest, sitagliptin

## Abstract

Blood glycosylated hemoglobin level can be affected by various factors in patients with type 2 diabetes and cardiovascular diseases. Frequent measurements are expensive, and a suitable estimation method could improve treatment outcomes.

**Patients and methods:**

93 patients were recruited in this research. We analyzed a number of parameters such as age, glucose level, blood pressure, Body Mass Index, cholesterol level, echocardiography et al. Patients were prescribed metformin. One group (n=60) additionally was taking sitagliptin. We applied eight machine learning methods (k nearest neighbors, Random Forest, Support Vector Machine, Extra Trees, XGBoost, Linear Regression including Lasso, and ElasticNet) to predict exact values of glycosylated hemoglobin in two years.

**Results:**

We applied a feature selection approach using step-by-step removal of them, Linear Regression on remaining features, and Pearson’s correlation coefficient on the validation set. As a result, we got four different subsets for each group. We compared all eight Machine Learning methods using different hyperparameters on validation sets and chose the best models. We tested the best models on the external testing set and got R^2^ = 0.88, C Index = 0.857, Accuracy = 0.846, and MAE (Mean Absolute Error) = 0.65 for the first group, R^2^ = 0.86, C Index = 0.80, Accuracy = 0.75, and MAE = 0.41 for the second group.

**Conclusion:**

The resulting algorithms could be used to assist clinical decision-making on prescribing anti-diabetic medications in pursuit of achieving glycemic control.

## 1 Introduction

Cardiovascular diseases and type 2 diabetes pose significant challenges to global healthcare systems, with a growing prevalence and associated morbidity and mortality rates (1). Achieving optimal glycemic control is crucial in managing type 2 diabetes and reducing the risk of cardiovascular complications. Glycosylated hemoglobin (HbA1c) is an important biomarker (2) that reflects the average blood glucose levels over a prolonged period, typically three months. Regular monitoring of HbA1c levels assists in assessing the effectiveness of treatment regimens and guiding therapeutic interventions. Hong et al. (3) provided a proof that high level of baseline HbA1c appeared to be an independent predictor for the severity of coronary artery disease (CAD) and poor outcome in patients with stable coronary artery disease.

Metformin and sitagliptin are commonly prescribed medications for the management of type 2 diabetes. These drugs have demonstrated efficacy in reducing HbA1c levels, but the individual response to treatment can vary significantly (4). Predicting HbA1c levels in patients with cardiovascular diseases and type 2 diabetes who are prescribed metformin and sitagliptin can aid in identifying patients at higher risk of poor glycemic control. By incorporating patient-specific factors, such as demographic characteristics, baseline HbA1c levels, comorbidities, and other factors, a predictive model can be developed to estimate HbA1c levels over a two-year follow-up period. This model can assist clinicians in optimizing treatment strategies and improving patient outcomes.

The primary objective of this study is to develop a predictive model for estimating HbA1c levels in patients with cardiovascular diseases and type 2 diabetes who were prescribed metformin and sitagliptin over a two-year follow-up period. The specific objectives include:

1. Collecting baseline demographic and clinical data, including age, gender, body mass index (BMI), duration of type 2 diabetes, baseline HbA1c levels, lipid profile, blood pressure, and other entries in anamnesis.
2. Utilizing statistical analysis and machine learning algorithms to identify significant predictors of HbA1c levels.
3. Developing a predictive model that incorporates patient-specific factors to estimate HbA1c levels over the two-year follow-up period.
4. Evaluating the performance of the predictive model using appropriate metrics, such as mean average error and R^2^.

## 2 Related work

Previous studies have investigated the prediction of HbA1c levels in patients with type 2 diabetes, but limited research has focused on patients with cardiovascular diseases and type 2 diabetes who are prescribed metformin and sitagliptin. Several predictive models have been developed using various machine learning algorithms, such as linear regression, decision trees, support vector machines, and artificial neural networks. These models have incorporated different sets of predictors, including demographic characteristics, clinical parameters, genetic markers, and lifestyle factors.

Tao et al. (5) built 16 machine learning models (Logistic regression, Decision Tree, Random Forest, Extra Tree, Stochastic Gradient Descent, Gaussian Naive Bayes, Bernoulli Naive Bayes, Multinomial Naive Bayes, Quadratic Discriminant Analysis, Linear Discriminant Analysis, Passive Aggressive, AdaBoost, Bagging, Gradient Boosting, XGBoost, and Ensemble Learning) to predict levels of HbA1c in a 3-month period. They included data on 100 000 patients to predict fasting blood glucose and over 2000 for Glycosylated Hemoglobin. XGBoost demonstrated AUC of 0.80 as the best model. Among most significant features they listed fasting blood glucose, body mass index (BMI), Age, Heart rate, alanine transaminase (ALT) and aspartate aminotransferase (AST), triglyceride (TG), metformin. However, their study did not include sitagliptin.

In another study by Wang et al. (6), a linear regression model was shown to be less effective in comparison with Random Forest, Support Vector Machines, and Neural Networks. They estimated HbA1c levels in patients with type 2 diabetes. Their model incorporated demographic characteristics, clinical parameters, and lifestyle factors. The model demonstrated good predictive performance, with an accuracy value of 0.73. After authors applied dimensionality reduction, the predictive accuracy rose to 0.75. Selected features included Hypertension, anamnesis, exercise, and total cholesterol (TC) as protective factors for HbA1c control. Central adiposity, family history, duration of type 2 diabetes mellitus, typical disease characteristics, complications, insulin dose, Oral hypoglycemic agents (OHA), fasting blood glucose (FBG), 2-hour postload blood glucose (2HBG), blood pressure, high-density lipoprotein-cholesterol (HDL-C), Low-density lipoprotein-cholesterol (LDL-C), and hypertension were risk factors for HbA1c control.

Five different machine learning algorithms were used by Fu et al. (7). They found statistically significant variables such as body mass index (BMI), pulse, Na, Cl, Alkaline Phosphatase (AKP). Among the used algorithms XGBoost had the highest accuracy of 78.18% and Area under the curve (AUC) value of 0.68 in testing set. They achieved a very high accuracy of 99% and AUC of 1 in training set but failed to produce same levels in testing set.

Wu et al. (8) built a model to predict Post-operative Acute Kidney Injury in patients based on Glycosylated Hemoglobin levels and other features. They demonstrated a link between kidneys and HbA1c that inspired inclusion of several blood test results related to kidneys in our study.

Zhang et al. (9) reviewed medical database to establish association between chronic and an increased risk for cardiovascular outcomes and all-cause mortality among patients with diabetes mellitus. Based on their research we selected a series of cardiovascular features into our study to represent that connection.

Research by Zeng et al. (10) demonstrated no significant difference between sitagliptin group and the control group on cardiovascular diseases in patients with type 2 diabetes mellitus. Thus, use of sitagliptin is not associated with an increased risk of poor outcomes in such patients.

While these studies provide valuable insights into predicting HbA1c levels in patients with type 2 diabetes, there is a need for research specifically focusing on patients with cardiovascular diseases and type 2 diabetes who are prescribed metformin and sitagliptin. This study aims to bridge this gap in the literature and develop a predictive model tailored to this population.

## 3 Materials and methods

### 3.1 Study design and participants

This prospective study aimed to develop a predictive model for estimating glycosylated hemoglobin (HbA1c) levels in patients with cardiovascular diseases (CVD) and type 2 diabetes (T2D) who were prescribed metformin and sitagliptin. Ethical approval was obtained from the institutional review board prior to study initiation, all patients signed the agreement to allow future use of their clinical data. Patients were prescribed medications based on medical standards by Ministry of Healthcare of Republic of Uzbekistan.

The study included a cohort of 93 patients of Republican scientific cardiology center diagnosed with both cardiovascular diseases and type 2 diabetes, aged 35-86 years, who received treatment with metformin or metformin and sitagliptin as part of their standard therapy. Patients with other forms of diabetes, such as type 1 diabetes or gestational diabetes, were excluded from the study. Additionally, patients with severe renal impairment (estimated glomerular filtration rate <30 mL/min/1.73m²) or significant liver dysfunction were excluded.

Our study aimed at predicting levels of glycosylated hemoglobin in patients with cardiovascular diseases and type 2 diabetes mellitus in a 2-year period based on the prescribed anti-diabetic medication: metformin and sitagliptin or just metformin. We performed detailed diagnostics of each patient in the study in the beginning and after two years. We recorded their anamnesis vitae, blood lab tests, Echocardiography results, prescribed medications, etc. All recordings were made both in the beginning and at the end of study. We tracked over 42 features and analysed their dynamics.

As the cost per patient of all tests is considerably higher than average income, the dataset was limited with only 93 patients. Additional 27 patients that took liraglutide were not included in this study.

### 3.2 Data collection

Baseline demographic and clinical data were collected from the participants at the start of the study. We included age (63.9 ± 8.8), gender (53.1% females), body mass index (BMI, 32.6 ± 6.4), duration of type 2 diabetes (7.3 ± 3.9 years), fasting blood glucose (10.07 ± 3.37 in the first group and 8.49 ± 2.79 in the second group), baseline HbA1c levels (9.34 ± 2.54 in the first group and 8.22 ± 2.10 in the second group), lipid profile (Total Cholesterol 184.19 ± 51.24, triglyceride 290.03 ± 304.80 in the first group and 197.12 ± 135.21 in the second group), blood pressure (139.35 ± 23.34 by 85.81 ± 11.99 in the first group, 140.61 ± 23.22 by 86.36 ± 13.22 in the second group), creatinine (93.5 ± 27.96), glomerular filtration rate (70.95 ± 19.63 in the first group, 63.22 ± 16.19 in the second group), ejection fraction (60.13 ± 6.79 in the first group, 56.47 ± 7.92 in the second group), presence of other comorbidities, and others. Over 30% of the patients had COVID-19 in their anamnesis by the end of the study.

HbA1c levels were measured at baseline and after the two-year follow-up period. Blood samples were collected using standard procedures and analyzed using high-performance liquid chromatography (HPLC) or a similar validated method.

### 3.3 Feature elimination

We eliminated all features that were missing in more than 10% cases. Then, we conducted a step-by-step procedure of excluding one feature, building a Linear Regression model on the training set, evaluating its performance on the validation set using Pearson’s correlation coefficient. Subsets with the highest performance values were selected. We continued the process until the correlation dropped below 95%.

The process was repeated for different objectives, such as glycosylated hemoglobin, fasting blood glucose, postprandial blood glucose, and ejection fraction.

### 3.4 Predictive model development

To develop the predictive model, various machine learning algorithms were utilized, including Random Forest, Support Vector Machine (SVM), XGBoost, Linear Regression (Least Squares, Lasso, ElasticNet), Extra Trees, and k nearest neighbors (k-NN). These algorithms were chosen based on their suitability for regression task, as well as their ability to handle complex and high-dimensional data in similar environment (7).

The dataset was randomly divided into training, validation, and external test sets in a ratio of 72:8:20. The training set was used to train the predictive models, while the validation set was used to evaluate their performance and perform hyperparameter tuning.

For each algorithm, a grid search approach was employed to optimize the hyperparameters. Cross-validation techniques, such as 10-fold cross-validation, were utilized to ensure robustness and prevent overfitting. The performance of each model was evaluated using various metrics, including mean absolute error (MAE), and R-squared value. We chose 10-fold validation as it provided sufficient data for training.

### 3.5 Model evaluation

The predictive models were evaluated using the validation set. The performance metrics, including mean absolute error (MAE), and R-squared value, were calculated to assess the accuracy and goodness-of-fit of each model. We selected the best model based on its performance on the validation set.

The best models were evaluated on the external test set afterwards. The validation test was not included in the final test as it was already used for best model selection and was no longer independent.

### 3.6 Statistical analysis

Descriptive statistics were used to summarize the baseline characteristics of the study participants. Continuous variables were presented as mean ± standard deviation (SD), range and interquartile range. Categorical variables were presented as frequencies and percentages.

Statistical analysis was performed using Python 3.7. Pearson’s correlations coefficient was used in feature selection process.

## 4 Results and discussion

### 4.1 Discussion of the importance of the selected features

Analysis of the dynamics of lipid spectrum indicators showed that the initial average values of cholesterol demonstrated the absence of a statistically significant difference between groups with different levels of HbA1c. However, there is a high positive correlation between postprandial glycose level and Metabolic Index (TG/HDL) (Kruskal test r=0.367; p=0.009) in patients with constantly decompensated HbA1 levels. The data obtained in our study allow us to consider HbA1c as a prognostically important indicator that allows us to retrospectively predict the lipid profile in patients with type 2 diabetes. Žďárská et al. (11) also found a significant correlation between dyslipidemia and postprandial glycemia (p = 0.013).

It is noteworthy that the main indicators of left ventricular diastolic pressure (LVDP) with a fairly high statistically significant power respond to therapy precisely in the group where 7.0<HbA1c<8.0. These indicators include a decrease in the time of isovolumetric relaxation for the left ventricular and right ventricular (Isovolumetric Relaxation Time (IVRT) ↓ Δ 4.5 (p = 0.008), the time of deceleration of early diastolic blood flow (early mitral flow deceleration time (DTE) ↓ Δ 20.12 (p = 0.01), the ratio of transmitral and transtricuspid velocity flows in early diastole to the speed of movement of the lateral part of the fibrous ring of the mitral and tricuspid valves (ratio between early mitral inflow velocity and mitral annular early diastolic velocity (E/e`) ↓ Δ 0.68 (p=0.08), as well as an increase in the speed of movement of the mitral valve ring in early diastole in the septal part e`septal ↑ Δ 0.97 (p=0.012), and, accordingly, its average e`average ↑Δ 0.52 (p=0.05). Negative dynamics of the e`average indicator were revealed in the groups HbA1c < 8 and HbA1c > 8 after 2 years of observation in contrast to the subgroups in which transitions in HbA1c were observed. The E/e` ratio in all groups showed a tendency to increase throughout the entire observation period, which indirectly indicates progression of diastolic dysfunction.

Ejection fraction, sizes of atriums and ventriculars, their filling velocities are included in the prediction of left ventricular diastolic dysfunction (LVDD). Chaudhary et al. (12) found HbA1C and age to be strong indicators of LVDD in newly diagnosed cases of Type 2 diabetes.

Patients with diabetes and hypertension belong to a group with very high cardiovascular risk according to Przezak et al. (13). Thus, blood pressure was found to be very important predictor of future development of cardiovascular diseases in patients with type 2 diabetes mellitus.

According to Hur et al. (14) Metformin can be used safely when the estimated glomerular filtration rate (eGFR) is ≥45 mL/min/1.73 m^2^. The range of eGFR in patients in our study was 37-100. If the eGFR is between 30 and 44 mL/min/1.73 m^2^ a daily dose of ≤1,000 mg is recommended to be continued. Therefore, close control of the state of kidneys’ function is very important when metformin is prescribed.

### 4.2 Predictive model performance on patients receiving both metformin and sitagliptin

Among the patients who received both metformin and sitagliptin, the Extra Trees algorithm exhibited the best performance in predicting glycosylated hemoglobin (HbA1c) levels. On the validation set, the Extra Trees model achieved an R-squared value of 0.5967 and a mean absolute error (MAE) of 0.997. These metrics indicated a moderate level of accuracy in predicting HbA1c levels over the two-year follow-up period. Statistical data on the selected features is demonstrated in **Table 1**.

**Table 1:** Statistical description of selected features used in the best model to predict glycosylated hemoglobin (HbA1c) in patients who took both metformin and sitagliptin.

Feature	Mean ± Standard deviation	Range	Interquartile range
Systolic blood pressure	139.4 ± 23.3	100 – 230	120 – 150
Diastolic blood pressure	85.8 ± 11.99	60 – 140	80 – 90
Fasting Blood Glucose	10 ± 3.4	5 – 21	8 – 11.5
HbA1c	9.3 ± 2.5	5 – 15.5	7.35 – 10.5
Cholesterol	184.2 ± 51.2	88 – 311	152 – 211.5
Ejection fraction	60 ± 6.8	45 – 71	57.2 – 65
Right Atrium size	32.2 ± 3.5	21 – 38	31 – 34
Right ventricular basal part	31.7 ± 3.9	18 – 39	30 – 34
Right ventricular central part	31.2 ± 3.1	20 – 38	30 – 33
Early mitral flow deceleration time (DTE)	209 ± 36.8	79 – 284	204 – 230.8

Furthermore, the Extra Trees model continued to demonstrate promising results when evaluated on the test set. It obtained a R-squared value of 0.88, C Index = 0.857, Accuracy of 0.846, and a MAE of 0.65, indicating good generalization performance. These findings suggest that the Extra Trees algorithm effectively captures the underlying patterns and relationships in the data, enabling accurate prediction of HbA1c levels in patients with cardiovascular diseases and type 2 diabetes who were prescribed both metformin and sitagliptin.

### 4.3 Predictive model performance on patients receiving only metformin

For patients who received only metformin, the Random Forest algorithm emerged as the best model in predicting HbA1c levels. On the validation set, the Random Forest model achieved an R-squared value of 0.3509 and a MAE of 0.6947, indicating a moderate level of accuracy. The model’s performance was further validated on the test set, where it achieved an R-squared value of 0.86, C Index of 0.8, Accuracy of 0.75, and a MAE of 0.41. These results demonstrate the ability of the Random Forest algorithm to effectively predict HbA1c levels in patients receiving only metformin. Statistical data on the selected features for this model is demonstrated in **Table 2**.

**Table 2:** Statistical description of selected features used in the best model to predict glycosylated hemoglobin (HbA1c) in patients who took only metformin.

Feature	Mean ± Standard deviation	Range	Interquartile range
Systolic blood pressure	140.6 ± 23.2	90 – 200	120 – 160
Fasting Blood Glucose	8.5 ± 2.8	4.5 – 17	6.8 – 8.7
Postprandial Blood Glucose	11.6 ± 4.3	5 – 20.8	8.2 – 14.9
HbA1c, %	8.2 ± 2.1	5 – 14	6.4 – 9.4
Glomerular Filtration Rate	63.2 ± 16.2	37 – 100	50 – 74
Ejection fraction	56.5 ± 7.9	42 – 71.9	48.6 – 62
Late (A) ventricular filling velocity	79.3 ± 13.0	42 – 100	75 – 88
Isovolumetric Relaxation Time (IVRT)	107.6 ± 23.7	65 – 145	92 – 135

### 4.4 Comparison with other models

The results of this study demonstrate the effectiveness of machine learning algorithms in predicting glycosylated hemoglobin (HbA1c) levels in patients with cardiovascular diseases and type 2 diabetes who were prescribed metformin and sitagliptin. In comparison to the Extra Trees and Random Forest models, the other algorithms, including Support Vector Machine (SVM), XGBoost, Linear Regression, and k nearest neighbors, underperformed on the validation set as shown in **Table 3**. These models exhibited lower R-squared values and higher MAE values, indicating a weaker predictive performance in estimating HbA1c levels in patients with cardiovascular diseases and type 2 diabetes who were prescribed either both metformin and sitagliptin or only metformin.

**Table 3:** Performance of the best models after their hyperparameter tuning on the validation set.

Machine Learning Algorithm	Medication	Performance of the best model on the validation set	
		MAE	R^2^
Random Forest	Metformin and Sitagliptin	1.0014	0.602
	Metformin	**0.3509**	**0.6947**
Support Vector Machine	Metformin and Sitagliptin	1.398	0.117
	Metformin	0.506	0.3674
Extra Trees	Metformin and Sitagliptin	**0.997**	**0.5967**
	Metformin	0.3666	0.685
k Nearest Neighbors	Metformin and Sitagliptin	1.2067	0.236
	Metformin	0.8611	-1.699
Least Squares	Metformin and Sitagliptin	1.279	-0.325
	Metformin	0.7129	-0.187
Lasso	Metformin and Sitagliptin	1.295	-0.0695
	Metformin	0.4280	0.5634
Elastic Net	Metformin and Sitagliptin	1.334	-0.1068
	Metformin	0.3978	0.6480
XGBoost	Metformin and Sitagliptin	1.0862	0.5879
	Metformin	0.699	-0.165

The smallest values of MAE and the largest values of R2 are in bold.

These findings suggest that the Extra Trees and Random Forest algorithms are better suited for predicting HbA1c levels in patients with cardiovascular diseases and type 2 diabetes who were prescribed either both metformin and sitagliptin or only metformin.

Our models outperformed Tao et al.’s best model (5) that got ROC AUC of 0.8 while our C Index achieved 0.86 in the first group. Fu et al. (7) used XGBoost that is prone to overfitting. The contrast between performance of their model on training set (99%) and test set (68%) demonstrates overfitting of the training process. Use of simple tree models such as Random Forest and Extra Trees helps fighting such a problem as each tree uses only subset of features.

Wang et al. (6) did not use ROC AUC to measure their performance. Accuracy of their model was 0.75 which is the same as accuracy of our model in the second group. However, our first group’s model achieved accuracy of 0.85.

### 4.5 Clinical implications

Accurate prediction of HbA1c levels in patients with cardiovascular diseases and type 2 diabetes is crucial for optimizing treatment strategies and improving long-term glycemic control. The findings of this study provide valuable insights into the use of machine learning algorithms for predicting HbA1c levels in patients prescribed metformin and sitagliptin. The Extra Trees and Random Forest models, in particular, demonstrate promising performance in estimating HbA1c levels over a two-year follow-up period. These models can assist clinicians in identifying patients at higher risk of poor glycemic control, enabling personalized treatment interventions and potentially reducing the risk of cardiovascular complications.

## 5 Conclusion

The results of this study highlight the effectiveness of machine learning algorithms, specifically the Extra Trees and Random Forest models, in predicting glycosylated hemoglobin (HbA1c) levels in patients with cardiovascular diseases and type 2 diabetes who were prescribed metformin and sitagliptin. The Extra Trees model demonstrated good performance in patients receiving both medications, while the Random Forest model was effective for patients receiving only metformin. These models can contribute to personalized treatment strategies and improved long-term glycemic control in this patient population. However, further research and validation in larger cohorts are warranted to confirm the findings and enhance the clinical applicability of these predictive models.

## Data availability statement

The raw data supporting the conclusions of this article will be made available by the authors, without undue reservation.

## Ethics statement

The studies involving humans were approved by Shek Aleksandr Borisovich, Professor at Republican Specialized Scientific Cardiology Center. The studies were conducted in accordance with the local legislation and institutional requirements. The participants provided their written informed consent to participate in this study.

## Author contributions

AI: Methodology, Writing – original draft. SM: Data curation, Investigation, Resources, Writing – original draft. RT: Project administration, Supervision, Validation, Writing – original draft. DA: Data curation, Methodology, Resources, Writing – review & editing. SA: Conceptualization, Formal Analysis, Writing – review & editing.
